# Transposable Element Dynamics among Asymbiotic and Ectomycorrhizal *Amanita* Fungi

**DOI:** 10.1093/gbe/evu121

**Published:** 2014-06-12

**Authors:** Jaqueline Hess, Inger Skrede, Benjamin E. Wolfe, Kurt LaButti, Robin A. Ohm, Igor V. Grigoriev, Anne Pringle

**Affiliations:** ^1^Department of Organismic and Evolutionary Biology, Harvard University; ^2^Section for Genetics and Evolutionary Biology, University of Oslo, Norway; ^3^FAS Center for Systems Biology, Harvard University; ^4^U.S. Department of Energy Joint Genome Institute, Walnut Creek, California

**Keywords:** evolution of symbiosis, genome architecture, phylogeny, repetitive DNA, ecological genomics

## Abstract

Transposable elements (TEs) are ubiquitous inhabitants of eukaryotic genomes and their proliferation and dispersal shape genome architectures and diversity. Nevertheless, TE dynamics are often explored for one species at a time and are rarely considered in ecological contexts. Recent work with plant pathogens suggests a link between symbiosis and TE abundance. The genomes of pathogenic fungi appear to house an increased abundance of TEs, and TEs are frequently associated with the genes involved in symbiosis. To investigate whether this pattern is general, and relevant to mutualistic plant-fungal symbioses, we sequenced the genomes of related asymbiotic (AS) and ectomycorrhizal (ECM) *Amanita* fungi. Using methods developed to interrogate both assembled and unassembled sequences, we characterized and quantified TEs across three AS and three ECM species, including the AS outgroup *Volvariella volvacea*. The ECM genomes are characterized by abundant numbers of TEs, an especially prominent feature of unassembled sequencing libraries. Increased TE activity in ECM species is also supported by phylogenetic analysis of the three most abundant TE superfamilies; phylogenies revealed many radiations within contemporary ECM species. However, the AS species *Amanita thiersii* also houses extensive amplifications of elements, highlighting the influence of additional evolutionary parameters on TE abundance. Our analyses provide further evidence for a link between symbiotic associations among plants and fungi, and increased TE activity, while highlighting the importance individual species’ natural histories may have in shaping genome architecture.

## Introduction

Transposable elements (TEs) are autonomously replicating pieces of DNA inhabiting the genomes of most life forms. The numbers of TEs encoded in species’ genomes vary widely, but bases coding for TEs often outnumber the protein-coding portion of a genome and can be as much as 85% of genomic DNA, for example in the maize strain B73 (Schnable et al. 2009). Because they lack any apparent function, TEs have classically been considered as junk DNA or genomic parasites (Doolittle and Sapienza 1980; Orgel and Crick 1980; Hickey 1982). However, during the last decade, ideas on the roles of TEs have changed, especially because of the increasing numbers of genomic sequences available that have highlighted the ability of TEs to generate genomic variation (e.g., Kidwell and Lisch 2001; Biémont 2010; Werren 2011; Hua-Van et al. 2011; but see McClintock 1983; Finnegan 1989 for earlier discussions). TEs are now more often described as commensal structural components of a genome, which can behave on a spectrum between parasitism and mutualism (Kidwell and Lisch 2001).

Two major classes of TEs can be distinguished, based on their modes of proliferation: Class I elements use an RNA-intermediate and move via a “copy-and-paste” mechanism. They include the long terminal repeat (LTR) elements and the long interspersed nuclear elements (LINE) (Finnegan 1989; Wicker et al. 2007). Class II elements transpose via DNA intermediates and can be further divided into subclasses depending on whether they use a “cut-and-paste” mechanism, like the terminal inverted repeat elements, or a “copy-and-paste” mechanism, for example the Helitrons (Kapitonov and Jurka 2001). Intact TEs encode the protein-coding sequences required for their proliferation, and upon activation can generate tens or hundreds of nearly identical copies that insert into new locations in the genome at varying degrees of specificity (reviewed in Levin and Moran 2011). By inserting themselves into or near coding genes, TEs can create loss of function mutations (Nekrutenko and Li 2001), confer new regulatory interactions through TE-encoded transcription factor binding sites (Jordan et al. 2003) or cause repeat-associated silencing of chromosomal neighborhoods (Hollister and Gaut 2009). Furthermore, high copy-number dispersed repeats can catalyze large-scale genomic rearrangements including inversions, duplications, deletions, and chromosomal translocations through recombination of nonallelic homologous TE insertions (Sen et al. 2006; Han et al. 2007; Robberecht et al. 2013).

TEs were at first thought to be relatively rare in fungi, presumably due to the small numbers found in genetic models, such as *Saccharomyces cerevisiae* and *Neurospora crassa*. However, genome sequencing efforts have revealed a wealth of TEs in a large diversity of fungal genomes (Daboussi and Capy 2003; Novikova et al. 2009; Muszewska et al. 2011). Plant pathogens often possess especially large, repeat-rich genomes (Raffaele and Kamoun 2012). This trend is most evident in biotrophic fungi with narrow host ranges, including, for example, the rice blast fungus *Magnaporthe grisea* (Dean et al. 2005), the oilseed rape pathogen *Leptosphaeria maculans* (van de Wouw et al. 2010), the powdery mildew *Blumeria graminis* (Spanu et al. 2010), and the leaf rust fungi *Puccinia graminis* and *Melampsora larici-populina* (Duplessis et al. 2011). There are, however, some exceptions to the pattern, for example the corn smut *Ustilago maydis* (Kämper et al. 2006), which has a relatively contracted and repeat-poor genome. Effectors, avirulence genes and other pathogenicity-related factors often cluster in repeat-rich regions and there are numerous examples implicating TE-mediated mechanisms in the genomic changes causing altered virulence or host-specificity (Kang et al. 2001; Sacristán et al. 2009; van de Wouw et al. 2010; Xue et al. 2012). These observations imply that the deleterious impacts of TEs may be negligible compared with the benefits provided by the increased genome plasticity conferred by TEs in the context of a host-pathogen coevolutionary arms race (Raffaele and Kamoun 2012).

The symbiosis of ectomycorrhizal (ECM) fungi and plants is also a biotrophic interaction, but functions as a mutualism; however, the mechanisms enabling symbiosis may be similar across the different kinds of associations (Veneault-Fourrey and Martin 2011). An ECM fungus grows with plant roots and provides various benefits to the plant in exchange for carbon (Smith and Read 2010). When the mutualism is established, gene expression programs are altered to enable the fungus to colonize root surfaces and grow between plant cells (Martin 2007). The formation of the symbiotic interface requires the fungus to communicate with the plant immune system, and the fungus may use tools comparable to host recognition mechanisms used by pathogens. For example, in the symbiosis between the ECM fungus *Laccaria bicolor* and the deciduous broadleaf tree *Populus trichocarpa*, an effector-like small secreted protein, *MiSSP7*, is secreted by the fungus and imported into the plant nucleus, where it directly modulates gene expression (Plett et al. 2011).

The genomes of the ECM fungi *L. bicolor* and *Tuber melanosporum* suggest that ECM genomes may also house elevated numbers of TEs. For example 60% and around 21–24% of the *T. melanosporum* and *L. bicolor* genomes, respectively, constitute TE-derived sequence (Martin et al. 2008, 2010; Labbé et al. 2012). ECM fungi coevolving with their hosts may experience selective pressures similar to those experienced by plant pathogens. Like pathogens, ECM fungi are obligately dependent on plants and the decline of one host species may necessitate the switch to another (Raffaele and Kamoun 2012). This dynamic may favor the maintenance of genome plasticity (Martin and Selosse 2008; Veneault-Fourrey and Martin 2011). However, a key assumption of the host-pathogen coevolutionary arms race model (Raffaele and Kamoun 2012) does not hold; in contrast to most biotrophic pathogens, many ECM fungi are generalists (Bruns and Bidartondo 2002; Kennedy et al. 2003; but see Smith et al. 2009) and an individual fungus associates with multiple trees (Horton and Bruns 2001; Saari et al. 2005).

**Fig. 1.—:**
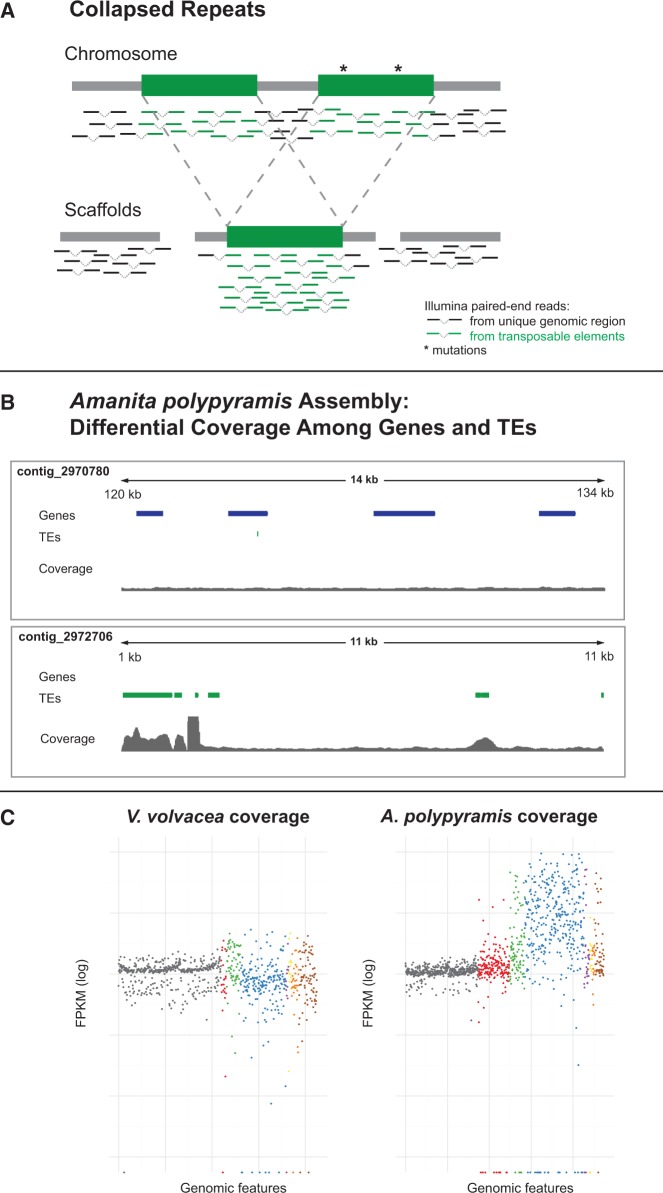
The challenges associated with estimating TE content from assemblies generated using short read data. (*A*) Assemblers cannot disambiguate reads from different locations and so collapse nearly identical repeats, often causing breakpoints in the assembly. (*B*) TE regions (green) on the *Amanita polypyramis* contig in the bottom panel show greatly increased coverage compared with the rest of the contig and the contig containing housekeeping (CEGMA) genes (blue, top panel), evidence of collapsed repeats. (*C*) Example of genome-wide coverage data for *Volvariella volvacea* (AS) and *A. polypyramis* (ECM). Gray points correspond to CEGMA genes and the points for transposable elements are colored by superfamily (see fig. 2). In *V. volvacea* TE coverage is within range of CEGMA coverage, whereas a large increase in the coverage of various elements, including for example Gypsy elements (blue), is visible in the *A. polypyramis* data.

Our current understanding of TE dynamics in ECM fungi is patchy and largely limited to comparisons between a small number of species (Labbé et al. 2012) or over large evolutionary distances (Novikova et al. 2009; Muszewska et al. 2011), making it difficult to comment on potential mechanisms shaping TE content. To investigate TE content evolution in ECM fungi at a finer resolution, we sequenced the genomes of five species of fungi within the genus *Amanita*, as well as the asymbiotic (AS) outgroup *Volvariella volvacea*. The genus *Amanita* encompasses more than 500 species, including the charismatic *A. muscaria* (often depicted in fairy tales) and the deadly poisonous death cap, *A. phalloides*. The genus is found on all continents and houses both ECM and free-living fungi. The number of symbiotic species, which associate with a diversity of plants, is far greater than the number of AS species. Furthermore, the AS *Amanita* have recently been shown to form a monophyletic clade basal to the ECM *Amanita*, supporting a single origin of ECM symbiosis within this genus (Wolfe, Tulloss, et al. 2012). We chose to sequence one representative from each of three large ECM clades: *A. brunnescens*, *A. polypyramis* and *A. muscaria* var. *guessowii*, as well as the AS species *A. thiersii* and *A. inopinata*. We developed analytical approaches to characterize and quantify TE content by combining assembly-based and assembly-free methods. The latter technique addresses the issue of underrepresentation of repeats in de novo assemblies derived from short sequencing reads (fig. 1) (Alkan et al. 2011).

We found ECM genomes to house elevated TE contents compared with *A. inopinata* and the outgroup *V. volvacea*, especially after consideration of unassembled reads. Results mirror the phylogenetic analyses of TE families, where large amplifications of TEs are found in ECM species. But, the AS species *A. thiersii* also houses a large number of TEs that have recently expanded.

## Materials and Methods

### Fungal Strains and DNA Extraction

Sources and cultures of *Amanita* and the outgroup species are described in table 1. Cultures were maintained on solid modified MMN medium (0.5 ml/l CaCl_2_[×2H_2_O], 0.5 ml/l FeCl_2_[×6H_2_O], 1 ml/l NaCl, 1 ml/l MgSO_4_[×7H_2_O], 5 ml/l [NH_4_]_2_HPO_4_, 10 ml/l KH_2_PO_4_, 2 g/l malt extract, 5 g/l potato dextrose broth, 5 g/l dextrose, 2 g/l cellobiose, 2 g/l polypeptone peptone, and 1 g/l yeast extract) with the addition of 100 × BME vitamins (MP Biomedicals, Santa Ana, CA) and antibiotics (150 mg/l streptomycin, 150 mg/l penicillin). For DNA extraction, fungi were grown on liquid modified MMN medium and incubated in the dark at 27°C for 2 weeks prior to harvesting. Harvested mycelia were ground in liquid nitrogen and extracted as described below.

**Table 1:** Fungal Strains

Species	Strain	Collector	Provenance	Date Collected	Niche	Habitat
*Amanita brunnescens*	Koide BX004	R. Koide	Haugh West, Pennsylvania	August 2003	ECM	With red pine
*A. polypyramis*	BW_CC	B. Wolfe (through Boston Mycological Club	Cape Cod, Massachusetts	October 2007	ECM	Mixed oak and pine forest
*A. muscaria*^a^	Koide BX008	R. Koide	Haugh West, Pennsylvania	August 2003	ECM	With red pine
*A. inopinata*	Kibby_2008	G. Kibby and B. Wolfe	Suffolk, United Kingdom	October 2008	AS	At edge of pasture
*A. thiersii*^b^	Skay4041	S. Kay	Baldwin City, Kansas	2009	AS	Lawn
*Volvariella volvacea*	PS #WC 439	Penn State Spawn Collection	China	1984	AS	Unknown

Note.—ECM, ectomycorrhizal.

^a^*Amanita muscaria* is a name used for a species complex (Geml et al. 2008); strain Koide BX008 is *A. muscaria* var. *guessowii* (www.amanitaceae.org, last accessed June 17, 2014).

^b^Wolfe, Kuo, et al. (2012).

*Amanita thiersii* DNA was extracted using the Qiagen genomic tip extraction protocol as per manufacturers’ instructions (Qiagen, Valencia, CA). DNA from the additional species was extracted using the “Phytophtora genomic DNA” phenol/chloroform protocol available from JGI (http://jgi.doe.gov/collaborate-with-jgi/pmo-overview/protocols-sample-preparation- information/, last accessed June 17, 2014). Following extraction, all samples were cleaned using Qiagen Genomic-tip 100/G columns, according to the manufacturers’ protocols and starting after the DNA isolation step (Qiagen, Valencia, CA). Quantity and quality of the samples were assessed using an Agilent 2100 Bioanalyzer.

### Sequencing and Assembly of JGI Genomes

The *A. thiersii* genome was sequenced using the Roche 454 and Illumina platforms including one 454 Rapid library, one 4-kb 454 paired-end library and one 2 × 76 3-kb Illumina paired-end library. An initial assembly of the Illumina data was generated using Velvet (Zerbino and Birney 2008), followed by a Newbler assembly of the resulting contigs together with the 454 libraries (-fe reads2remove -info -ace -qo -sio -a 50 -l 350 -g -ml 30 -mi 97). This resulted in a 45 × coverage assembly with 2,370 scaffolds, 36-kb scaffold N50, 37.2-Mb total scaffold, 5,969 contigs, 21.8-kb contig N50, and 39.4-Mb total contig. Allpaths fragment and jumping libraries were simulated from the Newbler contigs using wgsim (Li et al. 2009) with the following options: -e 0 -d 4000 -N 45000000 -1 100 -2 100 -r 0 -R 0 -X 0. The simulated and Illumina data were subsequently assembled with AllPathsLG release version R38445 (Gnerre et al. 2011), resulting in the assembly detailed in table 2.

**Table 2:** Draft Genome Assemblies

	ECM	ECM	ECM	ECM	AS	AS	AS
	*Amanita brunnescens*	*A. polypyramis*	*A. muscaria*	*A. muscaria*	*A. inopinata*	*A. thiersii*	*Volvariella volvacea*
			JGI			JGI	
Total assembly size (Mb)	57.6	23.5	40.7	67.6	22.1	33.7	52.4
Ploidy	Dikaryon	Dikaryon	Dikaryon	Dikaryon	Dikaryon	Monokaryon	Dikaryon
Assembler	ABySS	ABySS	AllpathsLG	ABySS	ABySS	AllpathsLG	ABySS
Number of scaffolds	17,039	5,295	1,011	17,516	5,912	1,446	4,019
Longest scaffold (kb)	497.0	384.1	1,491.6	158.6	2,165.3	1,038.0	1,066.4
Scaffold N50 (kb)	11.0	61.2	168.1	12.1	156.2	77.0	54.6
Number of contigs	24,844	6,690	3,814	24,994	6,157	2,164	6,360
Longest contig (kb)	260.6	384.2	508.8	158.6	2,081.7	1,038.0	719.7
Contig N50(kb)	8.6	48.5	30.1	10.5	86.6	60.4	44.0
CEGMA completeness %	94.6	95.6	92.3	92.3	96.0	96.0	95.6
CEGMA redundancy	1.8	1.3	1.1	2.9	1.1	1.1	1.7

Note.—Summary statistics of the draft genome assemblies generated for each species. Columns marked “JGI” highlight genomes assembled by DOE-JGI. ECM and AS refer to ectomycorrhizal and asymbiotic ecology, respectively. Percentages of CEGMA core eukaryotic genes (Parra et al. 2007) recovered in each assembly were used as estimates of gene space completeness. CEGMA redundancy is the average copy number of single copy CEGMA genes detected in each genome and serves as an indicator of the amount of heterozygosity in an assembly.

The *A. muscaria* var. *guessowii* genome was sequenced using the Illumina platform with one 2 × 100 3.5 kb Illumina long fragment paired-end library, one 2 × 100 3-kb Illumina paired-end unamplified library and one 2 × 150 27-kb Illumina paired-end unamplified library. Each fastq file was QC filtered for artifact/process contamination and subsequently assembled with AllPathsLG release version R42328 with HAPLOIDIFY = True (Gnerre et al. 2011), resulting in the assembly detailed in table 2.

### Sequencing and Assembly of Additional Genomes

We sequenced a single lane of Illumina reads for each of the additional species as well as an independent replicate of the *A. muscaria* genome. Paired-end libraries of 300-bp total fragment size were prepared at the Harvard Biopolymers facility (www.genome.med.harvard.edu, last accessed June 17, 2014) using the Illumina TruSeq gDNA protocol (Illumina, Cambridge, UK) and sequenced to 100 bp on an Illumina HiSeq2000 instrument. The raw read data were preprocessed using Trimmomatic v.0.22 (Lohse et al. 2012) to remove any residual sequencing adapters and low quality sequences. Leading and trailing bases with quality scores less than Q28 were trimmed and a sliding window analysis across 5-bp windows was used to eliminate reads when the average quality dropped below Q18. After adapter removal and low-quality trimming, any sequences shorter than 50 bp were removed from each data set.

The trimmed libraries were assembled using ABySS v.1.3.3 (Simpson et al. 2009) with the following parameters: *j* = 8, *S* = 200–5,000, *l* = (k-mer - 20) and *n* = 10 for all k-mer values between 33 and 89. Contiguity statistics (longest scaffold and N50), were calculated for each assembly after any scaffolds shorter than 200 bp were removed. We also scored different assemblies for completeness and redundancy by probing for core eukaryotic genes using CEGMA (Parra et al. 2007). Final assemblies were chosen to maximize contiguity and completeness while minimizing redundancy (table 2).

### TE Identification and Classification

TEs were identified using a combination of homology-based methods, de novo detection of overrepresented sequences, and structure-based approaches. We first screened the genome assemblies for TE-derived sequences using tBLASTX v.2.2.25 + (Gish and States 1993) with translated protein-coding sequences from Repbase v.17.08 (Jurka et al. 2005). The search was run without sequence filtering at an *e* value threshold of 10^−^^15^. In addition to tBLASTX searches we ran the BLASTER suite (Quesneville et al. 2003) for de novo detection as well as LTRHarvest (Ellinghaus et al. 2008) for structure-based detection of TEs. The results of all three searches were fed into the REPET TEdenovo pipeline (Flutre et al. 2011) that we modified to run on an LSF cluster. Briefly, TEdenovo uses the programs Piler (Edgar and Myers 2003), GROUPER (Quesneville et al. 2003), and RECON (Bao and Eddy 2002) to cluster the TEs identified by the different methods and reconstruct a consensus for each group of matches. The Python scripts we developed for pipelining elements of the REPET pipeline on an LSF cluster are available on request from the corresponding author.

The reconstructed TE consensus sequences were deduplicated and classified into class, order, and superfamily using the REPET TEclassifier (Flutre et al. 2011). TEclassifier is based on matches with Repbase, the presence of key Pfam (Finn et al. 2006) domains (e.g., reverse transcriptase or transposase domains), and structural features such as long-terminal repeats or target site duplications. Clustering cutoffs for consolidating individual elements were set at 95% identity over 98% of the element length as those were determined to be the optimal parameters for a low redundancy database of TEs (Flutre et al. 2011). The automatic assignments were manually assessed to remove false positives and spurious matches and to resolve conflicting annotations. The fragmented and repetitive nature of our genome assemblies (table 2) has the potential to cause inflated numbers of false positive matches in de novo searches, and so we decided on the following stringent filtering criteria: A TE was only retained if it had a significant BLAST match (<10^−^^6^) with an element in Repbase or contained a TE-derived Pfam domain (as defined by the REPET-curated Pfam library). Any matches that had a significant hit (< 10^−^^3^) to a non-TE Pfam domain were removed from the library.

For the final annotation of TEs in each of the genomes, we combined all reconstructed elements into a single library and used it as an input library for RepeatMasker v. 3.30 (Smit et al. 2010). RepeatMasker was run using an alignment cutoff of 250 (-cutoff 250) and sensitive search (-s). The TE locations identified by RepeatMasker were deduplicated using MATCHER from the BLASTER package (Quesneville et al. 2003), and we retained only the match with highest sequence identity in cases of overlapping annotations. This nonredundant set of TE annotations was used for all further analyses.

### Coverage-Based Quantification of TEs

Genome assemblies based on short-read sequencing data commonly suffer from an underrepresentation of repeated sequences (Alkan et al. 2011; fig. 1). As the majority of our assemblies are based on Illumina short-read libraries we sought to specifically target this issue and provide a different perspective by calculating TE content from the unassembled libraries using a depth-of-coverage approach. First, we assume an approximately even sequencing coverage across each genome. By comparing the sequencing depth of TE sequences to sequencing depth of unique genomic sequences, we calculate a metric enabling us to estimate the entire TE content of a library, both ancient TEs and relatively more recent, undiverged TEs.

This relative coverage for TE regions was calculated by first aligning our Illumina gDNA libraries to their respective assemblies. In the analysis of *A. thiersii*, we used a 76-bp paired-end library generated by the JGI available in SRA under accession number SRR065673. Reads were aligned using Bowtie 2 (Langmead and Salzberg 2012) in end-to-end alignment mode, reporting only the best match for each read. Fragment counts for all genomic regions were calculated using HTSeq-count (www-huber.embl.de/users/anders/HTSeq/, last accessed June 17, 2014), discarding reads that map to multiple features. TE regions were scored using the deduplicated RepeatMasker annotations to count the number of fragments by repeat ID, meaning that if a TE was found in multiple genomic locations, total counts for a repeat ID can reflect read counts consolidated over several different scaffolds. Coverage of the CEGMA gene regions was calculated accordingly, taking into account all reads mapping between the start of the first and end of the last exon, including introns. To alleviate mapping artifacts due to the intrinsically repetitive nature of TE sequences we decided to calculate the approximate TE copy number at the superfamily-level, on the basis of different superfamilies being sufficiently divergent to avoid unspecific mapping. A scaling factor *S_t_* for each superfamily was estimated as the ratio of the sum of fragments mapped per kilobase per million reads aligned (FPKM) of all target repeat IDs belonging to a superfamily over the median FPKM of all CEGMA genes. The corrected TE content estimates for each superfamily were calculated by scaling the assembled TE content by its scaling factor *S_t_*.

### TE Family Clustering, Prediction of Protein-Coding Regions, and Phylogenetic Analysis

Clustering of elements into TE families was performed using USEARCH v. 5.0.144 (Edgar 2010) with the parameters –id 0.8 –queryfract 0.8 –rev –maxrejects 128, choosing the longest element for each family as the representative sequence. Annotations for all TEs were updated to reflect the lowest level of classification shared between the members of a given family.

We first predicted protein-coding sequences for all repeat IDs using Genewise (Birney et al. 2004) with the amino acid sequences of the five best BLASTX matches in Repbase as targets and allowing for the inclusion of stop codons. In some cases, the annotated TEs do not span the entire protein-coding sequence, especially in regions where TEs are nested or in close proximity to one another (data not shown). To obtain the most complete possible set of TE-derived protein-coding sequences, we therefore included a second search, using the protein-coding sequences predicted from the repeat IDs to identify TE protein-coding sequences in the genome assemblies directly. We screened each assembly against the predicted TE proteins using BLASTX with an *e* value cutoff of 10^−^^15^. Scaffold fragments encompassing the candidate locations plus an additional 500-bp upstream and downstream were excised from the assemblies and fed into Genewise, together with the matching query sequences to obtain individual protein predictions for each TEs (as above).

For the phylogenetic analyses of our three target element superfamilies (Copia, Gypsy, and LINE), amino acid sequences belonging to each superfamily were aligned using an iterative approach. We first aligned sequences of at least 500 amino acids, as those are expected to yield better alignments. Alignments were run using PAGAN (Löytynoja et al. 2012), a phylogeny-aware aligner. To improve alignments, we calculated ML guide trees from the first alignments using RAxML v. 7.7.5 (Stamatakis 2006) with a WAG+Γ model, and then repeated alignments with the new guide trees.

PAGAN also implements a guided placement algorithm that can align shorter sequence fragments into existing alignments of full-length sequences. We used this feature to align predicted proteins that were shorter than 500 amino acids into the full-length TE superfamily alignments. Sequences shorter than 100 amino acids were omitted from analyses as those tended to align poorly even in a guided alignment (data not shown). Starting from the root of the ML guide tree, we tagged the deepest nodes containing only elements from the same species with the name of that species. Each fragment was then aligned into the best-fitting node for its species. To avoid disjoint alignments of short sequences spanning different domains, we removed all fragments that did not overlap the reverse transcriptase region by at least 25%. Finally, weakly aligning regions were trimmed from alignments using trimAl (Capella-Gutiérrez et al. 2009) with the following parameters: -gt 0.1. The resulting amino acid alignments contained 1,168 positions in 1,071 sequences (LINE), 1,289 positions in 330 sequences (Copia), and 1,287 positions in 1,229 sequences (Gypsy).

We determined the best-fit model for amino acid analyses using ProtTest 3.2 (Guindon and Gascuel 2003; Darriba et al. 2011). The JTT model of evolution (Jones et al. 1992) with Γ-distributed rates (+Γ) and empirical amino acid frequencies (+F) performed best for all three superfamilies independent of the selection criterion. Amino acid trees were calculated using RAxML v. 7.7.5 (Stamatakis 2006; Stamatakis et al. 2008) with the JTT+Γ+F model. Bootstrapping (BS) analyses for each tree were performed using the fast BS algorithm implemented in RAxML (-f a), with an automated stopping criterion (-autoMRE). BS runs stopped after 350 replicates in the case of LINE and Copia and 450 for the Gypsy alignment. Ultrametric trees were estimated from the ML trees using PATHd8 (Britton et al. 2007) and rooted with the *V. volvacea* outgroup that minimizes duplications and losses as determined using Notung 2.6 (Chen et al. 2000) with default parameters.

## Results

### Draft Genomes

We sequenced the genomes of the ECM fungi *A. brunnescens*, *A. polypyramis*, and *A. muscaria* var. *guessowii* (hereafter referred to simply as *A. muscaria*), the closely related saprotrophs *A. inopinata* and *A. thiersii*, and the more distantly related outgroup *V. volvacea*. Sequencing and assembly of *A. thiersii* and *A. muscaria* were completed as part of the Department of Energy Joint Genome Institute (JGI) Community Sequencing Programs (CSP# 402019 and 403202, respectively) and were based on multiple libraries of short- and long-range paired-end Illumina reads, plus additional *A. thiersii* 454 libraries. The draft genomes of all other species, as well as a replicate of the *A. muscaria* genome, were sequenced and assembled using single short-range PE Illumina libraries (table 2).

De novo assembly from single Illumina libraries proved a successful strategy for reconstructing gene space, and on average 95% of conserved eukaryotic (CEGMA) genes were recovered from each genome (table 2). The numbers of CEGMA genes found in single-library assemblies are comparable to those recovered from the multilibrary JGI assemblies although, not surprisingly, the single-library assemblies are considerably more fragmented. This point is illustrated in a direct comparison between the two *A. muscaria* assemblies (table 2). The same CEGMA genes are present in both assemblies despite the greatly different levels of fragmentation: Scaffold N50 was 168 kb in the JGI assembly, compared with 12 kb in the single-library assembly. We also see an increased level of redundancy in some of the single-library assemblies, which we interpret as a reflection of the inability of the assembler to distinguish whether two highly similar genomic regions arose from a recent duplication, or constitute the two heterozygous haplotypes of the region in a diploid genome. The level of redundancy may thus serve as an indicator of the heterozygosity found in the respective dikarya. Redundancy is most pronounced in the *A. muscaria*, *A. brunnescens*, and *V. volvacea* assemblies. The *A. muscaria* single-library assembly has an average copy number of 2.9 for each CEGMA gene, compared with 1.1 in the JGI assembly. The *A. brunnescens* and *V. volvacea* assemblies are both approaching an average copy number of 2. Thus, the relatively larger assembly size for these species may be explained by heterozygosity in these diploid fungi, and the assembly of different alleles onto different contigs, rather than by extensive genome expansion. This is supported by the recent publication of a monokaryotic *V. volvacea* genome sequence with a total assembly size of 35.7 Mb (Bao et al. 2013), which compares with the 52.4 Mb of our dikaryon assembly in a proportion that is similar to the estimated CEGMA redundancy (1.5). Our current focus is to quantify TE content, and not to compare protein-coding genes, and we do not attempt gene prediction beyond the CEGMA genes. Future publications will more formally compare the gene content of the different species.

### TE Prediction and Quantification Based on Assemblies

TEs were predicted from assembled genomes in two steps: First, we identified and reconstructed consensus elements in each assembly following the first part of the REPET pipeline (Flutre et al. 2011). The resulting single-species libraries were combined into an aggregate TE library (supplementary table S1 and data file S1, Supplementary Material online), and although it includes elements found in *V. volvacea*, for simplicity we refer to it as the “*Amanita* TE library” hereafter. Consensus elements were classified using the REPET classifier and manually filtered to remove individual elements where there was no direct evidence for identity as a TE (see Materials and Methods for details). Our approach risks discarding previously uncharacterized types of TEs, but with the limitations of our data in mind, we focused on tracking the dynamics of known families of TEs rather than exhaustively describing the complete set of TEs in any particular genome. For this reason, we also avoided a kmer-based analysis of repeat content.

The final *Amanita* TE library consists of 7,376 consensus elements belonging to 16 different superfamilies and includes all of the orders of TEs described in Wicker et al. (2007), with the exception of Crypton elements (supplementary fig. S1 and table S1, Supplementary Material online). A large proportion of the reconstructed TEs belong to the Gypsy and Copia superfamilies of LTR retroelements (51% and 18%, respectively), as is commonly found across the fungi (Daboussi and Capy 2003; Muszewska et al. 2011). Another large proportion of consensus elements (15%) belong to the LINE non-LTR retroelements. Together, class I elements make up over 80% of the *Amanita* TE library whereas a diversity of class II DNA tranposons only makes up about 15 % of the library. Clustering elements into families according to the “80–80–80” rule (80% of nucleotide identity over 80% of the sequence for at least 80 bp; Wicker et al. 2007) revealed 3,204 families with 2.3 members on average (supplementary table S1, Supplementary Material online).

**Fig. 2.—:**
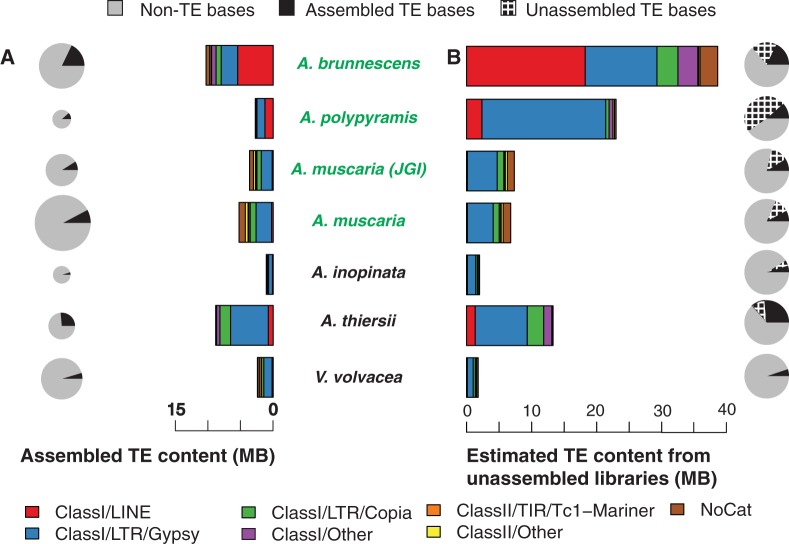
(*A*) TE content identified in draft genome assemblies. Pie charts show the percentage in each assembly annotated as TE (black) and non-TE (gray). Charts are scaled by overall assembly size. (*B*) Rescaled TE content based on relative coverage between TE and housekeeping genes (see Materials and Methods). Pie charts show the percentages of reads mapped to TE (black) and non-TE regions (gray). Darker gray sections denote the difference between unassembled and assembled data. Names of ectomycorrhizal species are marked in green, AS species in black.

The second step of our protocol used RepeatMasker (Smit et al. 2010) and the *Amanita* TE library to identify the location of individual repeats in each of our genome assemblies. Genomic regions that were annotated with more than one element were deduplicated, keeping only the best TE match (supplementary tables S2–S8, Supplementary Material online). Proportions of TEs found in draft assemblies varied from around 5% in *A. inopinata* and *V. volvacea* to 26% in *A. thiersii* (fig. 2*A*). Despite considerable differences in overall TE content, all of the species house a diverse set of TEs spanning most major superfamilies, although there are also low frequency repeats, for example the Maverick and Penelope elements, which show a more patchy distribution (supplementary fig. S1, Supplementary Material online). Generally, TE content in each genome mirrors the composition of the consensus library, with Gypsy and Copia superfamilies dominating TE populations. A large expansion of LINE is apparent in the genome of *A. brunnescens*, and to a lesser degree is also visible in its closest relative, *A. polypyramis*. A similar expansion, but of Gypsy elements, is evident in *A. thiersii*. Although the diversity (presence or absence) of elements is similar across all species, the relative frequencies of individual TE superfamilies are highly variable and show distinct amplification profiles.

### TE Quantification from Unassembled Libraries

A pitfall of whole genome shotgun (WGS) sequencing is the inability to accurately resolve nearly identical repeats in these data (Alkan et al. 2011; fig. 1). Read lengths and short-range library sizes are often shorter than an average TE, resulting in the superposition of TEs and other recently duplicated regions in WGS assemblies (fig. 1*A*). The median consensus length of complete elements reconstructed in *A. thiersii*, the only assembly in which we could identify a sizeable number of complete consensus elements, is 6,583 bp. That length is far larger than the 300-bp fragment size libraries used to sequence and assemble the single-library genomes. TE content estimates based on assembled draft genomes (fig. 2*A*) are likely to represent lower bounds. Estimates may also be biased toward more ancient TE insertions, which would have had time to accumulate mutations and will more easily resolve into separate scaffolds. Moreover, genome assemblies derived from diploid fungi will vary in the degree to which TE insertions that are present in both haplotypes have been assembled onto the same or different scaffolds. Heterozygous copies of the same TE insertion in a diploid genome may assemble onto different scaffolds. The degree to which this happens is unknown, but is likely to be different for each species. TE estimates from assembled content are not likely to be directly comparable (table 2).

Protocols to characterize TE content from raw sequencing libraries may obviate these issues and have been used effectively with plant genomes (Tenaillon et al. 2011; Hertweck 2013; Senerchia et al. 2013). To establish a different and perhaps more realistic picture of TE content, one that is comparable across species, we turned to the unassembled libraries and developed a sequencing coverage-based method to re-estimate the amount of TEs present in each genome (see Materials and Methods).

Our approach identified many TEs not found within the assembled genomes, confirming the presence of collapsed TE sequences in our assemblies and providing a different perspective on TE content across the phylogeny (fig. 2*B*). We found particularly large amounts of unassembled TEs in *A. brunnescens* and *A. polypyramis*, increasing the overall TE content estimated in these species to 36% and 59%, respectively. Although many different types of unassembled TEs are found in the genome of *A. brunnescens*, a distinct amplification of Gypsy elements is found in *A. polypyramis*. This amplification was already apparent in the raw coverage data (fig. 1*C*). Remaining species house moderate amounts of unassembled TEs, with the exception of *V. volvacea*, where coverage of TE regions tends to be lower than that of unique genomic sequence. This is likely an effect of ploidy; although the majority of CEGMA genes appear to be present as a single haplotype, and thus are mapped at higher coverage, the bulk of the TE regions appear to be present as either two haplotypes or only present on one of the chromosomes, and so are mapped at half the coverage (fig. 1*C*).

### Phylogenetic Analyses

To provide a phylogenetic perspective on our comparative data, and document patterns of amplification and loss of TE families, we analyzed the assembled portion of our TE repertoires in a phylogenetic framework. Protein sequences spanning the reverse transcriptase domains of the three largest superfamilies (Copia, LINE and Gypsy) were predicted from the genome assemblies, aligned and used to estimate maximum-likelihood (ML) phylogenies. Ultrametric trees for each superfamily were derived from ML trees by running a mean path length method (Britton et al. 2007).

**Fig. 3.—:**
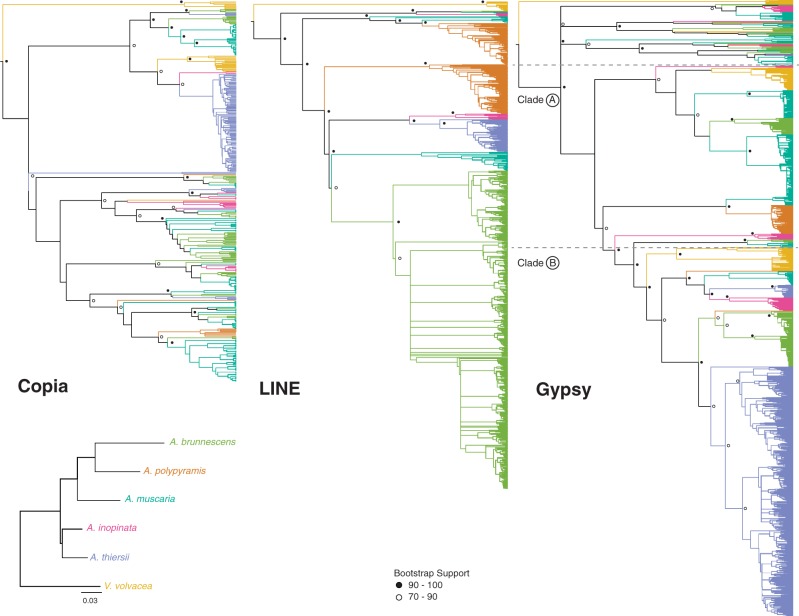
ML phylogenies of the predicted protein sequences of the three largest TE superfamilies. Branches are colored according to the species phylogeny shown bottom left (Wolfe, Tulloss, et al. 2012). Nodes near the root are marked according to their bootstrap support (circle: 70–90, filled circle: > 90).

The three superfamilies show contrasting phylogenetic patterns (fig. 3). The most pronounced differences are in the age distributions of the TE copies. Around half of the Copia elements belong to deep clades containing small numbers of elements from multiple species. The largest expansion is found in *A. thiersii* with 85 extant elements. In contrast, around 80% of LINE and Gypsy elements are part of young, species-specific clades, often encompassing hundreds of elements, for example the *A. brunnescens* expansion in LINE (699 elements) or the *A. thiersii* expansion in Gypsy elements (494 elements). These patterns imply that many of the Copia elements found in our genomes are derived from ancient amplifications, and that there was comparatively little recent activity, whereas the LINE and Gypsy superfamilies are characterized by abundant recent amplifications.

The phylogenetic data mirror patterns suggested by the comparative analysis of assembled TE content (fig. 2*A*). *Amanita thiersii*, the species with the highest assembled TE content, shows amplifications in all three superfamilies (fig. 3, blue clades). The most prominent amplification is found among Gypsy elements, where 494 elements (about 40% of the Gypsy elements analyzed) fall into a single *A. thiersii*-specific clade, whereas the *A. thiersii* clades among LINE and Copia amplifications are smaller (71 and 85 elements, respectively). Similarly, the large increase in the numbers of LINE seen in *A. brunnescens* and *A. polypyramis* reflects amplifications in these species (fig. 3, green and orange clades, respectively). *Amanita brunnescens* houses the largest clade with 699 elements, whereas *A. polypyramis* LINE have expanded in two separate clades containing 108 and 91 elements, respectively. Although *A. brunnescens* and *A. polypyramis* are close relatives and a common origin of the amplified LINEs seems plausible, our phylogenetic data suggest independent amplifications in *A. brunnescens* and *A. polypyramis*. The elements fall into distinct, strongly supported clades with bootstrap values between 97 and 100.

Gypsy elements show the most diverse patterns of TE activity. Species-specific amplifications are evident for all species, suggesting recent activity of Gypsy elements across the genus. We are able to distinguish at least five deep clades that predate the divergence of *V. volvacea* and the genus *Amanita*. TE amplifications are concentrated in two of these clades, marked clade A and clade B (fig. 3). Apart from a smaller amplification in *V. volvacea* (45 elements), clade A is dominated by ECM species which contribute 84% of the 356 extant elements. Within clade A we find three well-supported lineages that date to at least the base of the ECM species. Clade B houses TEs from a more diverse set of species and contains the large *A. thiersii* amplification discussed above, as well as a sizeable *A. brunnescens* amplification (110 elements).

### TE Amplification and ECM Ecology

Our different analyses provide distinct perspectives on TE proliferation and abundance in symbiotic fungi. Analyses based on assembled genomes suggest the AS, decomposer fungus *A. thiersii* as the species with the greatest proportion of TEs relative to coding sequence (TEs are 26% of the genome, fig. 2*A*), and although the genome of the ECM species *A. brunnescens* is also rich in TEs (18% of the genome), the ECM species *A. polypyramis* and *A. muscaria* house relatively modest proportions of repeats (11% and 9%, respectively). However, both *A. polypyramis* and *A. muscaria* house around twice as many TEs than either of the AS species *A. inopinata* or *V. volvacea* (5% in both species). Analyses based on unassembled genomes reveal a complementary pattern. Estimates of TE content in the ECM species are between two and five times greater than estimates based on assembled content (36% in *A. brunnescens*, 59% in *A. polypyramis*, and 22% in *A. muscaria*). The proportions of unassembled TE content found in the AS species were generally smaller, with almost no change in *V. volvacea* (5% total content), and about one and a half times as much in *A. inopinata* and *A. thiersii* (8% and 36% total TE content, respectively). Data suggest an excess of young, unassembled TE copies in several species, and most obviously in the ECM species.

All three superfamily phylogenies, but especially those of LINE and Gypsy elements (fig. 3) show the hallmarks of TE expansions in ECM species. By contrast, amplifications in either *A. inopinata* or *V. volvacea* are relatively modest and less frequent. Phylogenetic data suggest that different clades of TEs may have amplified independently in different ECM species, for example among LINE where the large *A. brunnescens* amplification groups with smaller clades from *A. muscaria*, *A. thiersii* and *A. inopinata*, rather than with the amplifications in its closest relative *A. polypyramis*. *Amanita brunnescens* and *A. muscaria* elements are also abundant among the TEs retained over longer evolutionary distances, as evident from their ample presence in the deeply divergent clades of the Copia and Gypsy superfamilies. The pattern of increased retention may point toward lower rates of TE loss in these ECM species.

Nevertheless, ECM species are not the only species housing TE expansions. The saprotroph *A. thiersii* is a species with a high proportion of TEs in the genome, and expansions of all three superfamilies are apparent.

## Discussion

### Methodological Aspects

Short-read sequencing has rapidly emerged as a widely used method for the study of genome evolution. The decreased cost of sequencing coupled with advances in bioinformatics has resulted in a growing understanding of the mechanisms shaping the evolution of gene content and regulation from broad phylogenetic scales to the fine-grained resolution of populations. Although most analyses are focused specifically on gene space in the wider sense (including genes and noncoding regulatory sequences), TEs, which can play a major role in the reshaping of genomic architecture (e.g., Sen et al. 2006; Han et al. 2007; Robberecht et al. 2013), often quite literally fall between the cracks.

We developed two, complementary approaches to analyze TE diversity and dynamics using short-read sequencing across six fungal genomes. We first assembled draft genomes to identify TE families and built a reference set of elements for annotation of assembled genomes. We then developed a method to probe the unassembled portions of our libraries, by comparing the relatively different sequencing depths of identified TEs and annotated housekeeping genes. Inclusion of the coverage-based quantification dramatically increased the predicted TE content in many species, underscoring the importance of using assembly-free methods to gauge TE content. Recently, coverage-based approaches using raw sequencing reads have been used effectively for quantification of TEs in plants (Tenaillon et al. 2011; Hertweck 2013; Senerchia et al. 2013). In the aggregate, our methods provide promising new approaches for extracting information about TE distributions from unassembled data.

In our data, the difference between assembled and unassembled estimates of TE content was most extreme in *A. polypyramis*, where the proportion of reads aligning into TE regions was almost fivefold higher than the proportion of assembled bases annotated as TEs (59% and 12%, respectively). Although the differences between assembled and unassembled proportions of TEs were less dramatic in the remaining species, our estimates of TE content increased across the board when we analyzed unassembled genomes. Moreover, the predicted proportion of TEs in the *A. muscaria* JGI assembly doubled, suggesting that the issue of underestimating TEs may also be relevant for multilibrary assemblies that include long insert size paired-end reads. The *A. polypyramis* data further underscore that high assembly contiguity is not necessarily an indicator of a comprehensive assembly (table 2), but in this case may be the result of extensive clustering, and therefore lack of assembly, of TEs outside of protein-coding regions.

Using a coverage-based approach also mitigates potential artifacts from the analysis of a mix of diploid and haploid genome sequences. Whether or not homozygous TE insertions are assembled onto the same or distinct contigs is dependent on the degree of heterozygosity, which may vary among TE families and between genomes. As relative coverage considers the abundance of TE sequences compared with reference genes among the complete set of reads, it implicitly accounts for the effects of heterozygosity.

One obvious shortcoming of our approach is its inability to detect wholly novel types of TEs as we annotate only these sequences commonly recognized as TEs, nor can our approach identify TEs that remain completely unassembled. The characterization of entirely novel types of TEs may always necessitate very high quality genome sequences, where TEs can be confidently placed into unique genomic contexts to determine their full extent. Other issues include biases resulting from the mapping of highly repetitive regions (Treangen and Salzberg 2011) and biases inherent in the sequencing protocol, for example, GC bias (Dohm et al. 2008) and PCR amplification bias (Aird et al. 2011). We have addressed mapping biases by analyzing only one hit per sequenced fragment, and averaging coverage over TE superfamilies, on the basis that superfamilies are sufficiently diverged between each other to avoid nonspecific cross mapping.

Comparison of the final TE content predictions between the two *A. muscaria* assemblies (fig. 2) shows that, although our estimates should be considered approximate, we obtain proportions that are within 3% of each other by mapping the same read data to two entirely independent assemblies generated using different sequencing strategies. We believe that we are capturing the most important signal in the data, even in the assemblies derived from a single lane of Illumina HiSeq sequencing.

### TE Content Correlates with Ecology

A clear signature of TE activity in ECM species is evident in both contemporary (fig. 2*B*) and historical (fig. 3) patterns. The three ECM species appear to be at different stages of TE invasion. *Amanita brunnescens* and *A. polypyramis* show signs of recent and ongoing TE activity, as manifested by the large ratios of unassembled to assembled TE content (fig. 2). The data suggest the presence of large numbers of young TE insertions that are too similar to assemble onto different contigs. Recently active families were also suggested by the presence of large amplified clades, especially in LINE and Gypsy elements (fig. 3). In contrast, *A. muscaria* houses a more modest proportion of TEs. TEs may have proliferated less extensively in the *A. muscaria* genome. However, phylogenetic analyses provide evidence for a number of amplifications in *A. muscaria* (fig. 3), suggesting that *A. muscaria* has also experienced TE expansions at some point in the past, even if recent TE activity is less than it is in *A. brunnescens* or *A. polypyramis*.

The AS genomes of *V. volvacea* and *A. inopinata* demonstrate a very different pattern. These genomes encode low amounts of TEs, and we found only modest evidence of recent activity in either unassembled TE content or TE superfamily phylogenies. However, the signatures of TE activity found in *A. thiersii* are a stark contrast to *A. inopinata* and *V. volvacea*. The *A. thiersii* genome provided evidence for recent amplifications of all three superfamilies and harbored TE populations almost three times the size of the *V. volvacea* or *A. inopinata* genomes (figs. 2 and 3). These data challenge the simple association of an ECM niche with higher TE content in the *Amanita*.

The numbers of TE insertions residing in a genome are dependent on 1) the rate of transposition and 2) the rate of survival of TE copies (Charlesworth B and Charlesworth D1983). A number of ecological and population genetic processes influence rates of transposition and survival. The transposition rate is modulated by regulation of active TE copies. Among others, TEs may be activated by stress (Grandbastien 1998; Capy et al. 2000) or silenced by genome defense mechanisms (Daboussi and Capy 2003). TE survival depends on the impact an insertion has on the genome and, if it is deleterious, the ability of natural selection to remove it from the population before it is fixed. Small effective population sizes reduce the effectiveness of selection, allowing altered rates of fixation of deleterious TEs (Charlesworth B and Charlesworth D 1983; Lynch and Conery 2003). Demographic events, including population bottlenecks, may reduce the effective population size, resulting in slower rates of TE loss and consequentially higher rates of fixation (Gherman et al. 2007; Lockton et al. 2008). The mating system of the organism will also influence TE retention. In theory, the spread of a new TE copy across a population of selfing organisms is difficult and unlikely (Boutin et al. 2012). But, already established elements may be retained more readily, for example because of a potential reduction in the negative impact of ectopic recombination between dispersed TEs when insertions are homozygous (Montgomery et al. 1987; Boutin et al. 2012). Selfing also results in a decrease of the effective population size (Nordborg 2000).

Understanding patterns of TE distributions across a phylogeny and differentiating among the processes that drive patterns requires rich contextual information about species’ natural histories. *Amanita thiersii* is currently undergoing a range expansion in North America (Wolfe, Kuo, et al. 2012), and genetic diversity across its new range is very low, suggesting that the species is experiencing a population bottleneck and has a small effective population size. Data from other organisms suggest that this demographic scenario enables TE proliferation in Eukaryotes (Lynch and Conery 2003; Gherman et al. 2007; Lockton et al. 2008). A population bottleneck is also expected to similarly effect different classes of repeats (Gherman et al. 2007), which is consistent with our discovery that all three superfamilies we investigated show amplifications in *A. thiersii*.

A common narrative to explain TE expansions among the ECM species is less obvious. In contrast to the established link between pathogenicity effectors and TEs in plant pathogens (Sacristán et al. 2009; Rouxel et al. 2011), more evidence linking TEs with genes involved in the establishment and maintenance of symbiosis will be required to confirm that TEs enable genome flexibility and the symbiotic niche. Whether or not common mechanistic processes drive the expansions of TEs in ECM species, and if so, whether they are acting on the rate of transposition or rate of TE survival also remain to be determined.

Although the ECM *Amanita* fit patterns described for *L. bicolor* and *T. melanosporum* (Martin and Selosse 2008; Martin et al. 2010; Veneault-Fourrey and Martin 2011), there is no simple association between high TE content and the ECM niche. TEs directly influence host-specificity genes in plant pathogenic fungi (Sacristán et al. 2009; Rouxel et al. 2011), nonetheless additional forces may also influence increased TE abundance in plant pathogens. As demonstrated by the wide abundance of TEs in *A. thiersii*, the particular natural histories of species may also influence TE distributions. For example, among the biotrophic pathogens listed in the introduction, most have both sexual and asexual phases in their lifecycle (McDonald and Linde 2002; Giraud et al. 2008), a pattern shown to result in elevated number of TEs in cyclically sexual populations of *Daphnia pulex* (Schaack, Choi, et al. 2010; Schaack, Pritham, et al. 2010). A more detailed dissection of the different processes influencing TE insertion, dispersal, and survival is needed to disentangle the causal from the incidental and enable a holistic understanding of the adaptive impact of TEs in biotrophic fungi.

## Data Deposition

Raw sequencing libraries and assemblies for the *A. brunnescens*, *A. polypyramis*, *A. muscaria* (replicate), *A. inopinata*, and *V. volvacea* genomes have been deposited at National Center for Biotechnology Information (NCBI), BioProject numbers PRJNA236753, PRJNA236755, PRJNA236758, PRJNA236757, and PRJNA236756. The genome sequences of *A. muscaria* and *A. thiersii* are available at JGI (http://genome.jgi.doe.gov/programs/fungi/index.jsf, last accessed June 17, 2014) and associated data have been deposited at NCBI, BioProjects PRJNA207684 and PRJNA82749, respectively. The sequence alignments of TE families used for phylogenetic analysis are available from the corresponding author by request.

## Supplementary Material

Supplementary data file S1, figure S1, and tables S1–S8 are available at *Genome Biology and Evolution* online (http://www.gbe.oxfordjournals.org/).

Supplementary Data

## References

[evu121-B1] Aird D, Ross MG, Chen WS, Danielsson M (2011). Analyzing and minimizing PCR amplification bias in Illumina sequencing libraries. Genome Biol..

[evu121-B2] Alkan C, Sajjadian S, Eichler EE (2011). Limitations of next-generation genome sequence assembly. Nat Methods..

[evu121-B3] Bao D (2013). Sequencing and comparative analysis of the straw mushroom (*Volvariella volvacea*) genome. PLoS One.

[evu121-B4] Bao Z, Eddy SR (2002). Automated de novo identification of repeat sequence families in sequenced genomes. Genome Res..

[evu121-B5] Biémont C (2010). A brief history of the status of transposable elements: from junk DNA to major players in evolution. Genetics.

[evu121-B6] Birney E, Clamp M, Durbin R (2004). GeneWise and Genomewise. Genome Res..

[evu121-B7] Boutin TS, Le Rouzic A, Capy P (2012). How does selfing affect the dynamics of selfish transposable elements?. Mob DNA.

[evu121-B8] Britton T, Anderson CL, Jacquet D, Lundqvist S, Bremer K (2007). Estimating divergence times in large phylogenetic trees. Syst Biol..

[evu121-B9] Bruns TD, Bidartondo MI (2002). Host specificity in ectomycorrhizal communities: what do the exceptions tell us?. Integr Comp Biol..

[evu121-B10] Capella-Gutiérrez S, Silla-Martínez JM, Gabaldón T (2009). trimAl: a tool for automated alignment trimming in large-scale phylogenetic analyses. Bioinformatics.

[evu121-B11] Capy P, Gasperi G, Biémont C, Bazin C (2000). Stress and transposable elements: co-evolution or useful parasites?. Heredity.

[evu121-B12] Charlesworth B, Charlesworth D (1983). The population dynamics of transposable elements. Genet Res..

[evu121-B13] Chen K, Durand D, Farach-Colton M (2000). NOTUNG: a program for dating gene duplications and optimizing gene family trees. J Comput Biol..

[evu121-B14] Daboussi MJ, Capy P (2003). Transposable elements in filamentous fungi. Annu Rev Microbiol..

[evu121-B15] Darriba D, Taboada GL, Doallo R, Posada D (2011). ProtTest 3: fast selection of best-fit models of protein evolution. Bioinformatics.

[evu121-B16] Dean RA (2005). The genome sequence of the rice blast fungus *Magnaporthe grisea*. Nature.

[evu121-B17] Dohm JC, Lottaz C, Borodina T, Himmelbauer H (2008). Substantial biases in ultra-short read data sets from high-throughput DNA sequencing. Nucleic Acids Res..

[evu121-B18] Doolittle WF, Sapienza C (1980). Selfish genes, the phenotype paradigm and genome evolution. Nature.

[evu121-B19] Duplessis S (2011). Obligate biotrophy features unraveled by the genomic analysis of rust fungi. Proc Natl Acad Sci U S A..

[evu121-B20] Edgar RC (2010). Search and clustering orders of magnitude faster than BLAST. Bioinformatics.

[evu121-B21] Edgar RC, Myers EW (2003). PILER: Identification and classification of genomic repeats. Bioinformatics.

[evu121-B22] Ellinghaus D, Kurtz S, Willhoeft U (2008). LTRharvest, an efficient and flexible software for de novo detection of LTR retrotransposons. BMC Bioinformatics.

[evu121-B23] Finn RD, Mistry J, Schuster-Böckler B (2006). Pfam: clans, web tools and services. Nucleic Acids Res..

[evu121-B24] Finnegan DJ (1989). Eukaryotic transposable elements and genome evolution. Trends Genet..

[evu121-B25] Flutre T, Duprat E, Feuillet C, Quesneville H (2011). Considering transposable element diversification in de novo annotation approaches. PLoS One.

[evu121-B26] Geml J, Tulloss RE, Laursen GA, Sazanova NA, Taylor DL (2008). Evidence for strong inter- and intracontinental phylogeographic structure in *Amanita muscaria*, a wind-dispersed ectomycorrhizal basidiomycete. Mol Phylogenet Evol..

[evu121-B27] Gherman A (2007). Population bottlenecks as a potential major shaping force of human genome architecture. PLoS Genet..

[evu121-B28] Giraud T, Enjalbert J, Fournier E, Delmotte F, Dutech C (2008). Population genetics of fungal diseases of plants. Parasite.

[evu121-B29] Gish W, States DJ (1993). Identification of protein coding regions by database similarity search. Nat Genet..

[evu121-B30] Gnerre S (2011). High-quality draft assemblies of mammalian genomes from massively parallel sequence data. Proc Natl Acad Sci U S A..

[evu121-B31] Grandbastien M (1998). Activation of plant retrotransposons under stress conditions. Trends Plant Sci..

[evu121-B32] Guindon S, Gascuel O (2003). A simple, fast, and accurate algorithm to estimate large phylogenies by maximum likelihood. Syst Biol..

[evu121-B33] Han K (2007). Alu recombination-mediated structural deletions in the chimpanzee genome. PLoS Genet..

[evu121-B34] Hertweck KL (2013). Assembly and comparative analysis of transposable elements from low coverage genomic sequence data in Asparagales. Genome.

[evu121-B35] Hickey DA (1982). Selfish DNA: a sexually-transmitted nuclear parasite. Genetics.

[evu121-B36] Hollister JD, Gaut BS (2009). Epigenetic silencing of transposable elements: a trade-off between reduced transposition and deleterious effects on neighboring gene expression. Genome Res..

[evu121-B37] Horton TR, Bruns TD (2001). The molecular revolution in ectomycorrhizal ecology: peeking into the black-box. Mol Ecol..

[evu121-B38] Hua-Van A, Le Rouzic A, Boutin TS, Filée J, Capy P (2011). The struggle for life of the genome’s selfish architects. Biol Direct..

[evu121-B39] Jones DT, Taylor WR, Thornton JM (1992). The rapid generation of mutation data matrices from protein sequences. Comput Appl Biosci..

[evu121-B40] Jordan IK, Rogozin IB, Glazko GV, Koonin EV (2003). Origin of a substantial fraction of human regulatory sequences from transposable elements. Trends Genet..

[evu121-B41] Jurka J (2005). Repbase Update, a database of eukaryotic repetitive elements. Cytogenet Genome Res..

[evu121-B42] Kämper J (2006). Insights from the genome of the biotrophic fungal plant pathogen *Ustilago maydis*. Nature.

[evu121-B43] Kang S, Lebrun MH, Farrall L, Valent B (2001). Gain of virulence caused by insertion of a Pot3 transposon in a *Magnaporthe grisea* avirulence gene. Mol Plant Microbe Interact..

[evu121-B44] Kapitonov VV, Jurka J (2001). Rolling-circle transposons in eukaryotes. Proc Natl Acad Sci U S A..

[evu121-B45] Kennedy PG, Izzo AD, Bruns TD (2003). There is high potential for the formation of common mycorrhizal networks between understorey and canopy trees in a mixed evergreen forest. J Ecol..

[evu121-B46] Kidwell MG, Lisch DR (2001). Perspective: transposable elements, parasitic DNA, and genome evolution. Evolution.

[evu121-B47] Labbé J (2012). Characterization of transposable elements in the ectomycorrhizal fungus *Laccaria bicolor*. PLoS One.

[evu121-B48] Langmead B, Salzberg SL (2012). Fast gapped-read alignment with Bowtie 2. Nat Methods..

[evu121-B49] Levin HL, Moran JV (2011). Dynamic interactions between transposable elements and their hosts. Nat Rev Genet..

[evu121-B50] Li H (2009). The Sequence Alignment/Map format and SAMtools. Bioinformatics.

[evu121-B51] Lockton S, Ross-Ibarra J, Gaut BS (2008). Demography and weak selection drive patterns of transposable element diversity in natural populations of *Arabidopsis* lyrata. Proc Natl Acad Sci U S A..

[evu121-B52] Lohse M (2012). RobiNA: a user-friendly, integrated software solution for RNA-Seq-based transcriptomics. Nucleic Acids Res..

[evu121-B53] Löytynoja A, Vilella AJ, Goldman N (2012). Accurate extension of multiple sequence alignments using a phylogeny-aware graph algorithm. Bioinformatics.

[evu121-B54] Lynch M, Conery JS (2003). The origins of genome complexity. Science.

[evu121-B55] Martin F, Howard R, Gow N (2007). Fair trade in the underworld: the ectomycorrhizal symbiosis. Biology of the fungal cell.

[evu121-B58] Martin F, Selosse MA (2008). The *Laccaria* genome: a symbiont blueprint decoded. New Phytol..

[evu121-B56] Martin F (2008). The genome of *Laccaria bicolor* provides insights into mycorrhizal symbiosis. Nature.

[evu121-B57] Martin F (2010). Périgord black truffle genome uncovers evolutionary origins and mechanisms of symbiosis. Nature.

[evu121-B59] McClintock B (1983). The significance of responses of the genome to challenge. Science.

[evu121-B60] McDonald BA, Linde C (2002). The population genetics of plant pathogens and breeding strategies for durable resistance. Euphytica.

[evu121-B61] Montgomery E, Charlesworth B, Langley CH (1987). A test for the role of natural selection in the stabilization of transposable element copy number in a population of *Drosophila melanogaster*. Genet Res..

[evu121-B62] Muszewska A, Hoffman-Sommer M, Grynberg M (2011). LTR retrotransposons in fungi. PLoS One.

[evu121-B63] Nekrutenko A, Li WH (2001). Transposable elements are found in a large protein-coding genes. Trends Genet..

[evu121-B64] Nordborg M (2000). Linkage disequilibrium, gene trees and selfing: an ancestral recombination graph with partial self-fertilization. Genetics.

[evu121-B65] Novikova O, Fet V, Blinov A (2009). Non-LTR retrotransposons in fungi. Funct Integr Genomics..

[evu121-B66] Orgel LE, Crick FH (1980). Selfish DNA: the ultimate parasite. Nature.

[evu121-B67] Parra G, Bradnam K, Korf I (2007). CEGMA: a pipeline to accurately annotate core genes in eukaryotic genomes. Bioinformatics.

[evu121-B68] Plett JM (2011). A secreted effector protein of *Laccaria bicolor* is required for symbiosis development. Curr Biol..

[evu121-B69] Quesneville H, Nouaud D, Anxolabéhère D (2003). Detection of new transposable element families in *Drosophila melanogaster* and *Anopheles gambiae* genomes. J Mol Evol..

[evu121-B70] Raffaele SS, Kamoun SS (2012). Genome evolution in filamentous plant pathogens: why bigger can be better. Nat Rev Microbiol..

[evu121-B71] Robberecht C, Voet T, Esteki MZ, Nowakowska BA, Vermeesch JR (2013). Nonallelic homologous recombination between retrotransposable elements is a driver of de novo unbalanced translocations. Genome Res..

[evu121-B72] Rouxel T (2011). Effector diversification within compartments of the *Leptosphaeria maculans* genome affected by Repeat-Induced Point mutations. Nat Commun..

[evu121-B73] Saari SK, Campbell CD, Russell J, Alexander IJ, Anderson IC (2005). Pine microsatellite markers allow roots and ectomycorrhizas to be linked to individual trees. New Phytol..

[evu121-B74] Sacristán S (2009). Coevolution between a family of parasite virulence effectors and a class of LINE-1 retrotransposons. PLoS One.

[evu121-B75] Schaack S, Choi E, Lynch M, Pritham EJ (2010). DNA transposons and the role of recombination in mutation accumulation in *Daphnia pulex*. Genome Biol..

[evu121-B76] Schaack S, Pritham EJ, Wolf A, Lynch M (2010). DNA transposon dynamics in populations of *Daphnia pulex* with and without sex. Proc Biol Sci..

[evu121-B77] Schnable PS (2009). The B73 maize genome: complexity, diversity, and dynamics. Science.

[evu121-B78] Sen SK, Han K, Wang J, Lee J, Wang H (2006). Human genomic deletions mediated by recombination between Alu elements. Am J Hum Genet..

[evu121-B79] Senerchia N, Wicker T, Felber F, Parisod C (2013). Evolutionary dynamics of retrotransposons assessed by high throughput sequencing in wild relatives of wheat. Genome Biol Evol..

[evu121-B80] Simpson JT (2009). ABySS: a parallel assembler for short read sequence data. Genome Res..

[evu121-B81] Smit A, Hublery R, Green P (2010). http://www.repeatmasker.org.

[evu121-B82] Smith ME, Douhan GW, Fremier AK, Rizzo DM (2009). Are true multihost fungi the exception or the rule? Dominant ectomycorrhizal fungi on *Pinus sabiniana* differ from those on co-occurring *Quercus* species. New Phytol..

[evu121-B83] Smith SE, Read DJ (2010). Mycorrhizal symbiosis.

[evu121-B84] Spanu PD (2010). Genome expansion and gene loss in powdery mildew fungi reveal tradeoffs in extreme parasitism. Science.

[evu121-B85] Stamatakis A (2006). RAxML-VI-HPC: maximum likelihood-based phylogenetic analyses with thousands of taxa and mixed models. Bioinformatics.

[evu121-B86] Stamatakis A, Hoover P, Rougemont J (2008). A rapid bootstrap algorithm for the RAxML Web servers. Syst Biol..

[evu121-B87] Tenaillon MI, Hufford MB, Gaut BS, Ross-Ibarra J (2011). Genome size and transposable element content as determined by high-throughput sequencing in maize and *Zea luxurians*. Genome Biol Evol..

[evu121-B88] Treangen TJ, Salzberg SL (2011). Repetitive DNA and next-generation sequencing: computational challenges and solutions. Nat Rev Genet..

[evu121-B89] van de Wouw AP (2010). Evolution of linked avirulence effectors in *Leptosphaeria maculans* is affected by genomic environment and exposure to resistance genes in host plants. PLoS Pathog..

[evu121-B90] Veneault-Fourrey C, Martin F (2011). Mutualistic interactions on a knife-edge between saprotrophy and pathogenesis. Curr Opin Plant Biol..

[evu121-B91] Werren JH (2011). Colloquium Paper: selfish genetic elements, genetic conflict, and evolutionary innovation. Proc Natl Acad Sci U S A..

[evu121-B92] Wicker T (2007). A unified classification system for eukaryotic transposable elements. Nat Rev Genet..

[evu121-B93] Wolfe BE, Kuo M, Pringle A (2012). *Amanita thiersii* is a saprotrophic fungus expanding its range in the United States. Mycologia.

[evu121-B94] Wolfe BE, Tulloss RE, Pringle A (2012). The irreversible loss of a decomposition pathway marks the single origin of an ectomycorrhizal symbiosis. PLoS One.

[evu121-B95] Xue M (2012). Comparative analysis of the genomes of two field isolates of the rice blast fungus *Magnaporthe oryzae*. PLoS Genet..

[evu121-B96] Zerbino DRD, Birney EE (2008). Velvet: algorithms for de novo short read assembly using de Bruijn graphs. Genome Biol..

