# Supplementary material for: Transposable Element Dynamics among Asymbiotic and Ectomycorrhizal Amanita Fungi
Source: Genome Biol Evol. 2014 Jun 12;6(7):1564–78. doi: 10.1093/gbe/evu121 (PMC4122921; doi:10.1093/gbe/evu121)
Supplement: Supplementary Data [file supp_6_7_1564__index.html]

Transposable element dynamics among asymbiotic and ectomycorrhizal Amanita fungi — Transposable Element Dynamics among Asymbiotic and Ectomycorrhizal Amanita Fungi — Supplementary Data 

# Transposable Element Dynamics among Asymbiotic and Ectomycorrhizal *Amanita* Fungi

## Supplementary Data

files

**Files in this Data Supplement:**

- Supplementary Data - zip file
