# Supplementary figures and images for: Transposable Element Dynamics among Asymbiotic and Ectomycorrhizal Amanita Fungi
Source: Genome Biol Evol. 2014 Jun 12;6(7):1564–78. doi: 10.1093/gbe/evu121 (PMC4122921; doi:10.1093/gbe/evu121)

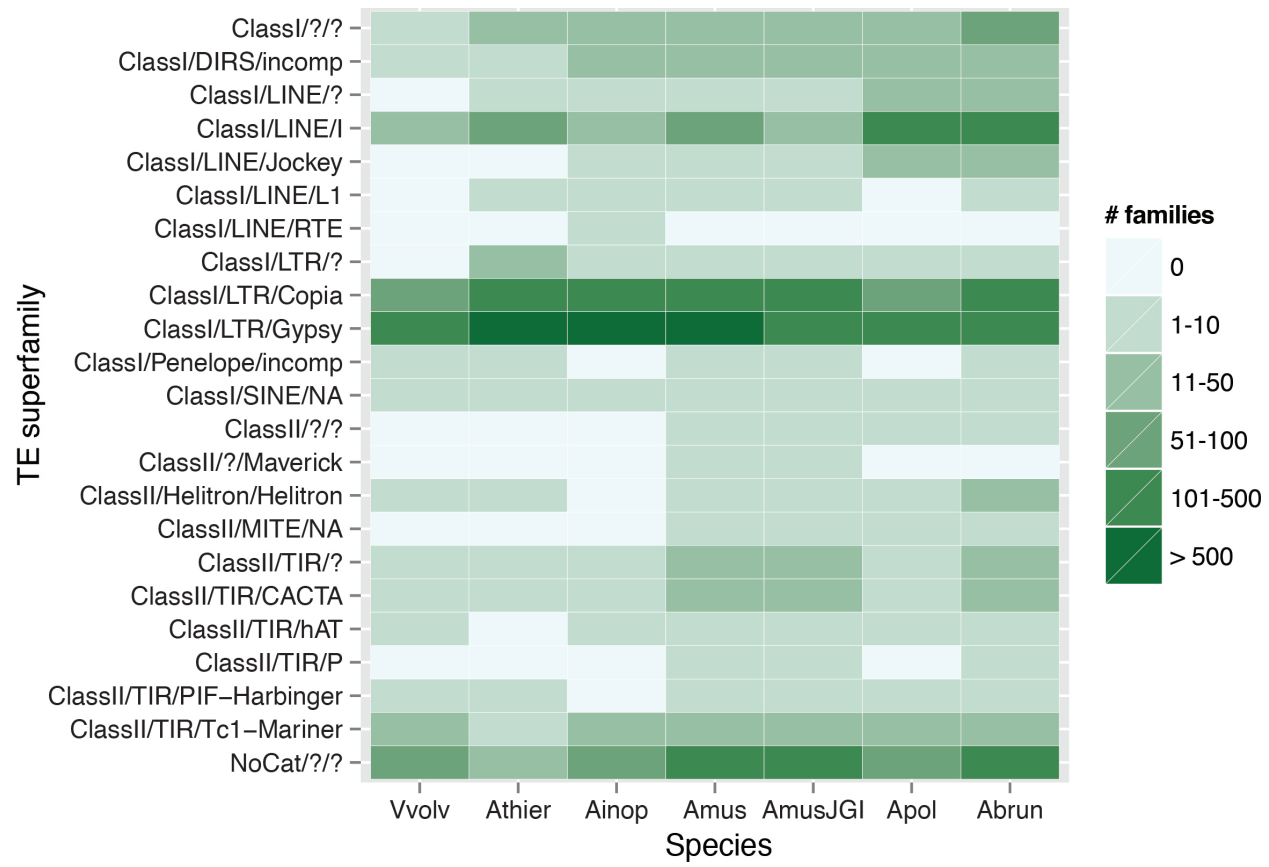

Figure 1: Distribution of TE superfamilies found in assembled genomes

Supplement: Supplementary Data [file supp_evu121_suppl_data.zip › SupplementaryMaterial.pdf]
